# Comparison of Efficacy in the Treatment of Otomycosis using 10% Povidone-Iodine and Clotrimazole Solution: Randomized, Double-Blind Control Trial

**DOI:** 10.1055/s-0045-1810001

**Published:** 2025-09-26

**Authors:** Pilasluck Numchaichanakij, Pattraporn Kreesaeng

**Affiliations:** 1 Department of Ear, Nose, and Throat, Chulabhorn Royal Academy, Bangkok, Thailand

**Keywords:** otomycosis, otitis externa, fungus, antifungal, topical eardrop

## Abstract

**Introduction:**

The main treatment of otomycosis has required the use of topical antifungal drops and/or acidifying agents. However, because of the availability issues of antifungal drops in developing countries, treatment with acidifying agents alone is necessary.

**Objective:**

The objective of this study was to compare the efficacy in the treatment of otomycosis using a 10% povidone-iodine and clotrimazole solution.

**Methods:**

Patients with otomycosis were assigned to 10% povidone-iodine group and Clotrimazole group. The patients were evaluated on the response of each group at 1, 2,4 and 8 weeks.

**Results:**

93 patients were assigned to the 10% povidone-iodine group and 95 patients to the Clotrimazole group. the response to treatment between groups. In the second week after treatment, 37.64% of the 10% povidone-iodine group and 40% of the Clotrimazole group had a good response to treatment (p = 0.551). In the fourth week after treatment, 52.69% of the 10% povidone-iodine group and 55.79% of the Clotrimazole group had a good response to treatment (p = 0.644). In the eighth week after treatment, 58.06% of the 10% povidone-iodine group and 64.21% of the Clotrimazole group had a good response to treatment (p = 0.642) In the 12th week after treatment, 76.34% of the 10% povidone-iodine group and 85.26% of the Clotrimazole group had a good response to treatment (p = 0.248).

**Conclusions:**

There were no significant differences in the response to between 10% povidone-iodine and Clotrimazole groups in the second, fourth, eighth and twelfths after treatment.

## Introduction

Otomycosis is a fungal infection of the external ear that is frequently encountered in otolaryngology clinics. It is one of the common causes of externa otitis (infection or inflammation of the external auditory canal), which physicians frequently encounter in otolaryngology clinics. Multiple factors are responsible for increasing the risk of otitis externa, including excessive moisture, foreign body in the ear canal, and any trauma, such as excessive cleaning or aggressive scratching of the ear canal. The most common cause of otitis externa is bacterial infection. 1 However, fungal infection accounts for 2 to 10% of cases of otitis externa and frequently occurs after treatment of bacterial infection. Other risk factors for otomycosis, including the immunocompromised host (e.g., diabetes mellitus), people who live in tropical climates, or people who swim frequently. 2

The most common signs and symptoms of otomycosis are pain, pruritus, discharge, and hearing loss. Otoscopic examination appears to be edematous and erythematous in the ear canal. The debris or fluid discharge is typically yellow, brown, white, or gray. The general appearance of the discharge may reveal the causative pathogen (e.g., fine, dark coating with Aspergillus; white, sebaceous-like material with Candida. 3 The main treatment of otomycosis has required the removal of cerumen, debris, and fungal elements from the external ear canal followed by the use of topical antifungal drops and/or acidifying agents. 4 5 6 Acidifying agents have been shown to successfully treat most mild cases of otomycosis, especially when combined with a thorough external canal debridement. 7 The advantage of topical antifungals combined with acidifying agents is that it absorbed on the surface of the ear canal skin with a higher concentration. 8 The use of antifungal solutions alone, such as clotrimazole or nystatin, are effective in the treatment of Candida infections, 9 10 however, in developing countries, topical antifungal drops may not be available, especially in rural areas. In that case, physicians may need to use only acidifying agents, such as 10% povidone-iodine, to treat otomycosis.

The objective of this study is to determine the treatment of otomycosis with acidifying agent compared with antifungal drop. We choose 10% povidone-iodine and Clotrimazole solution in our study because of the availability in Thailand, with the evidence of effectiveness. 8 If 10% povidone-iodine is effective as Clotrimazole solution, we can recommend the use of 10% povidone-iodine for the treatment of otomycosis patients in rural area, especially if the clotrimazole solution is not available.

## Methods

This double-blind randomized control trial approved by the Ethics Committee of Chulabhorn Research Institute (institutional review board no. 063/60). The inclusion criteria were as follows: patients who have signs and symptoms of otomycosis with confirmation of fungus by the KOH method and those who can participate in post-treatment follow-up as required. Patients who have a history of clotrimazole or 10% povidone-iodine allergy and a history of ear surgery were excluded. The ears examination was recorded on the quantity of secretion and the severity of inflammation before prescribing topical ear drop. The samples from the external ear were taken using a special speculum in a sterile container and sent to the laboratory. In the laboratory, the samples were evaluated using the KOH method and the presence of fungus characteristics was confirmed.

According to four blocks, the participants were assigned to the experimental (10% povidone-iodine) group and the control group (Clotrimazole). The 10% povidone-iodine group received 10% povidone-iodine ear drop twice daily and the Clotrimazole group received clotrimazole solution ear drop twice daily. The patients were examined 1, 2, 4 and 8 weeks after treatment by an otolaryngologist who did not know the type of treatment. The treatment response was classified into three groups based on clinical response: complete response (no external ear canal inflammation, no fungi and no secretion), partial response (decrease secretion, few fungal and mild inflammation), and nonresponse (hypersecretion with fungal and inflammation in the external ear canal). In the complete response group, the topical ear drop was discontinued, but follow-up continued in the recording form. If the nonresponders were considered in the eighth week, the resistant regimen was systemic antifungal treatment and an additional gentian violet drop into the ears. In the partial response group at eighth week, we will continue the assigned agent and reevaluate at twelfth weeks, the resistant regimen was systemic antifungal treatment and an additional gentian violet drop into the ears. After the twelfth week, we will discontinuation of follow up in patients with complete response. In partial response and nonresponse patients at twelfth weeks, we will make an appointment at an otolaryngology outpatient clinic in two weeks with continuation of systemic antifungal treatment and an additional gentian violet drop.

### Statistical Analyzes

Data was analyzed using the Social Sciences statistical package version 22 software (IBM Inc., Armonk, NY, USA). The baseline characteristics of the two groups were compared using descriptive statistics. The response to treatment two groups was compared using the independent sample t test and Fisher's exact test. p < 0.05 was considered significant.

## Results

### Baseline Characteristics

Between May 1, 2020, and July 31, 2021, 200 patients were enrolled in the study. In total, 93 patients were assigned to the 10% povidone-iodine group and 95 patients to the Clotrimazole group. Table 1 shows the demographic characteristics of the patients in both groups. The average age of the patients in the experimental group and the control group was 47.7 and 47.2 years. There were 45 male patients and 48 female patients in the 10% povidone-iodine group and 48 male patients and 47 female patients in the Clotrimazole group. There was no significant difference between the two groups.

**Table 1:** Demographic characteristics

Demographic characteristics	Betadine group	Clotrimazole group	p-value
Age (mean ± SD)	47.7 ± 18.33	47.2 ± 17.27	0.771
Gender (%) Male Female	45 (48.39) 48 (51.61)	48 (50.53) 47 (49.47)	0.769

Table 2 shows the response to treatment between groups. In the second week after treatment, 35 patients (37.64%) in the 10% povidone-iodine group and 38 patients (40%) in the Clotrimazole group had a good response to treatment (p = 0.551). In the fourth week after treatment, 49 patients (52.69%) in the 10% povidone-iodine group and 53 patients (55.79%) in the Clotrimazole group had a good response to treatment (p = 0.644). In the eighth week after treatment, 54 patients (58.06%) in the 10% povidone-iodine group and 61 patients (64.21%) in the Clotrimazole group had a good response to treatment (p = 0.642). The last follow-up on the twelfth week after treatment, 71 patients (76.34%) in the 10% povidone-iodine group and 81 patients (85.26%) in the Clotrimazole group had a good response to treatment (p = 0.248). Hence, there was no significant difference in treatment response between 10% povidone-iodine and Clotrimazole groups in the second, fourth, eighth and twelfth weeks after treatment.

**Table 2:** Comparison of response to treatment

Course of treatment	Type of treatment	Response to treatment			p-value
		No response (%)	Partial response (%)	Good response (%)	
Week 2	10% povidone-iodine Clotrimazole	29 (31.18) 23 (24.21)	29 (31.18) 34 (35.79)	35 (37.64) 38 (40.00)	0.551
Week 4	10% povidone-iodine Clotrimazole	14 (15.05) 17 (17.89)	30 (32.26) 25 (26.32)	49 (52.69) 53 (55.79)	0.644
Week 8	10% povidone-iodine Clotrimazole	7 (7.53) 5 (5.26)	32 (34.41) 29 (30.53)	54 (58.06) 61 (64.21)	0.642
Week 12	10% povidone-iodine Clotrimazole	5 (5.38) 2 (2.11)	17 (18.28) 12 (12.63)	71 (76.34) 81 (85.26)	0.248

## Discussion

Otomycosis, a common condition encountered in any ENT practice, can often be a challenging and frustrating entity for both patients and treating otolaryngologists. The most common sign and symptom of otomycosis are itching and ear fullness. Although complications are rare and rarely life-threatening, they often recur and may require prolonged treatment and follow-up. In addition to medication, effective treatment includes cleaning up and removing fungus spores from the ear canal. The purpose of treatment is not only to cure the infection, but also to alleviate the symptoms and signs caused by this condition. The high recurrence is attributed to the persistence of the spores. Chronic suppurative otitis media, postoperative mastoidectomy cavities, and immunosuppressed individuals are well documented predisposing factors for this condition. Therefore, in our study we have excluded the above conditions and tried to analyze whether there are other predisposing factors to otomycosis.

We selected 10% povidone-iodine, an antiseptic, as it was easily available and has been proven to be effective in chronic suppurative otitis media, which is one of the predisposing factors of otomycosis. 8 It is chemically stable and inexpensive, and resistance to bacteria and fungi is yet to be reported. Excessive and indiscrete use of any topical antibiotic and antimicrobials may lead to the emergence of resistant organisms. 10% povidone-iodine solves this problem as to date there are no studies showing resistance development, which is a growing concern in this era. In developing countries like Thailand, where a cheaper and effective form of ototoxicity-free drug is required, 10% povidone-iodine is potentially an effective alternative treatment.

In general, in our study there was no statistically significant difference in terms of response to treatment in the second, fourth, eighth and twelfth after treatment between two treatment groups of 10% povidone-iodine and clotrimazole solution. Therefore, we conclude that 10% povidone iodine may be an alternative treatment, especially in the event of clotrimazole in short supply, which we have encountered periodically in Thailand. There is no incidence of newly developed allergic reaction to both clotrimazole and 10% povidone-iodine in our study. Since we excluded patients with a history of allergic reactions to both agents, it is plausible that no allergic reaction is encountered.

In this clinical study, the side effects of 10% povidone-iodine were a burning sensation in the ears, and the yellow color of 10% povidone-iodine can stain clothes, which are extremely difficult to remove. To help minimize the risk of apparent clothes staining, which may discourage patients from continuing treatment, we encouraged patients to wear dark color clothes or for family members to help apply ear drops. The benefit of 10% povidone-iodine is that it dries faster compared to clotrimazole. Treatment of otomycosis requires early detection and timely treatment of patients with respect to the possibility of drug resistance in chronic infections. It should be noted that the basis for the treatment of otomycosis is keeping the ear dry and the ear hygiene, and the 10% povidone-iodine treatment can make the ear cavity dry faster than clotrimazole. If otomycosis has a prolonged treatment lasting longer than 12 weeks, the protocol should be considered according to the different types of fungi that cause the disease and the sensitivity to different antifungal drugs. In that case, additional oral antifungals should be considered for the 10% povidone-iodine regimen.

Clotrimazole is a drug that contains azole groups and is used to treat infections. 10% povidone-iodine is also an easily accessible remedy for infections, and its effect has been proven on chronic suppurative otitis media as the precipitating factors of otomycosis. This drug is a stable, inexpensive substance, and resistance to bacterial and fungal agents has not been reported yet. In our study, we found that 68.82% and 75.79% of patients in the 10% povidone-iodine and clotrimazole group, respectively, are at least partially responding to treatment. For this reason, we recommend at least 2 weeks of treatment.

## Conclusion

In conclusion, the result of this study supports the use of 10% povidone-iodine in the treatment of otomycosis. It is a good choice for the treatment of otomycosis in developing countries, due to its low cost, availability, effectiveness, and lack of ototoxicity. Furthermore, the effectiveness of Clotrimazole combined with 10% povidone-iodine, compared with Clotrimazole alone is an interesting subject that we would like to explore in the due time.
